# GLUT1-DS Brain Organoids Exhibit Increased Sensitivity to Metabolic and Pharmacological Induction of Epileptiform Activity

**DOI:** 10.3390/ph19010105

**Published:** 2026-01-07

**Authors:** Loïc Lengacher, Sylvain Lengacher, Pierre J. Magistretti, Charles Finsterwald

**Affiliations:** GliaPharm SA, 1202 Geneva, Switzerland

**Keywords:** GLUT1-DS, brain organoid, epilepsy, glucose, hypometabolism, hyperexcitability, iPSCs, astrocyte, neuron

## Abstract

**Background/Objectives**: Glucose Transporter 1 Deficiency Syndrome (GLUT1-DS) is a neurodevelopmental disorder caused by mutations in the gene encoding glucose transporter 1 (GLUT1), which leads to impaired glucose transport into the brain and is characterized by drug-resistant epilepsy. Limited glucose supply disrupts neuronal and astrocytic energy homeostasis, but how hypometabolism translates into network hyperexcitability remains poorly understood. Here, we used induced pluripotent stem cells (iPSCs)-derived brain organoids to examine how reduced metabolic substrate availability shapes epileptiform dynamics in human neuronal circuits from GLUT1-DS. **Methods**: Brain organoids were generated from a healthy donor or a GLUT1-DS patient and interfaced with multielectrode arrays (MEA) for recording of neuronal activity. A unified Python (v3.10)-based analytical pipeline was developed to quantify spikes, bursts, and power spectral density (PSD) across frequency bands of neuronal activity. Organoids were challenged with reduced glucose, pentylenetetrazol (PTZ), potassium chloride (KCl), and tetrodotoxin (TTX) to assess metabolic and pharmacological modulation of excitability. **Results**: GLUT1-DS organoids exhibited elevated baseline hyperexcitability compared to healthy control, characterized by increased spike rates, prolonged bursts, increased spikes per burst, and elevated PSD. Reduced glucose availability further amplified these features selectively in GLUT1-DS. **Conclusions**: Human brain organoids reproduce the pathological coupling between hypometabolism and hyperexcitability in GLUT1-DS. Our platform provides a mechanistic model and quantification tool for evaluating metabolic and anti-epileptic therapeutic strategies.

## 1. Introduction

Glucose Transporter 1 Deficiency Syndrome (GLUT1-DS) is an orphan neurodevelopmental disorder caused by mutations in slc2a1, the gene encoding for the Glucose Transporter 1 (GLUT1) protein that mediates glucose transport across the blood–brain barrier [1,2,3,4]. Mutations, which reduce GLUT1 expression or trafficking in GLUT1-DS reduce cerebral glucose availability, producing chronic brain hypometabolism and clinical symptoms including motor dysfunction, cognitive impairment, and drug-resistant epilepsy. Because the brain relies almost exclusively on glucose as its primary energy substrate, reduced glucose uptake disrupts the maintenance of ion gradients, neurotransmitter recycling, synaptic plasticity and neuroprotective processes [5,6]. This metabolic impairment also destabilizes the balance between excitation and inhibition, rendering neuronal networks unstable and susceptible to epileptic synchronization [7,8,9,10].

Astrocytes play a central role in this metabolic interplay. They express high levels of GLUT1 and act as metabolic intermediaries between the vasculature and neurons, linking cerebral blood flow to neuronal activity [11,12]. In the brain, GLUT1 is primarily expressed in astrocytes and endothelial cells at the blood–brain barrier. Astrocytes elaborate cytoarchitecture is composed of fine processes forming discrete domains that contact both synapses and capillaries, which position them as key sensors of extracellular signals and metabolic needs. At synapses, astrocytic processes express glutamate transporters that detect neuronal activity, while their vascular endfeet express GLUT1 to mediate glucose uptake from the capillaries. When entering astrocytes, glucose is predominantly metabolized through aerobic glycolysis to produce lactate, which is then exported via monocarboxylate transporter 1 (MCT1) and MCT4 [6]. Neurons, in turn, import lactate through MCT2, converting it to pyruvate that enters the tricarboxylic acid cycle to generate ATP via oxidative phosphorylation in the mitochondria. This process, known as the astrocyte–neuron lactate shuttle (ANLS), enables neurons to offload glycolysis and directly use lactate over glucose as an efficient energy substrate upon activity [13,14,15]. The ANLS plays critical roles in neuroprotection, synaptic plasticity, and memory consolidation [6,16,17,18,19]. Reduced metabolic support of neurons is a hallmark of several neurological diseases including Alzheimer’s disease, Parkinson’s disease, amyotrophic lateral sclerosis, depression and GLUT1-DS [5,20].

When GLUT1 function is compromised, transport of glucose into the brain and its uptake by astrocytes is markedly reduced, causing collapse of the ANLS. Consequently, GLUT1-DS is characterized not only by diminished glucose levels in the brain, but also by reduced lactate concentrations in the cerebral spinal fluid [2,21]. Energy failure in astrocytes further disrupts their ability to clear extracellular glutamate and potassium, leading to their accumulation, neuronal depolarization, and network hyperexcitability [9]. Moreover, glutamatergic signaling has been shown to enhance membrane expression of GLUT1 in oligodendrocytes, strengthening metabolic support to axons during heightened neuronal activity [22,23]. Thus, impaired GLUT1 function disrupts not only endothelial glucose transport but also astrocytic and oligodendrocytic contributions to neuronal energy homeostasis, ultimately destabilizing the excitatory–inhibitory balance of cortical networks.

To investigate these mechanisms in a controlled human cellular context, we employed brain organoids derived from healthy volunteer or GLUT1-DS patient-induced pluripotent stem cells (iPSCs), as described previously [24]. These three-dimensional neural assemblies, which are composed of both neuronal and glial cells populations, recapitulate aspects of cortical development, enabling the study of cellular maturation, metabolic interactions, and network behavior. When interfaced with multi-electrode arrays (MEAs), organoids allow for real-time, non-invasive recording of neuronal activity [25,26,27,28,29]. Our recent advances on this platform combine dense microelectrode grids with a new analytical pipeline for quantifications of spikes, bursts, and power spectral densities (PSD) under defined genetic, metabolic and pharmacological conditions. This approach provides new analytical tools to bridge single-cell metabolism with organ-level electrophysiology, providing an experimental window into the consequences of energy deprivation on human neuronal networks, specifically in the case of GLUT1-DS.

## 2. Results

### 2.1. New Analytical Pipeline for Electrophysiological Measurements in Brain Organoids

**Figure 1:**
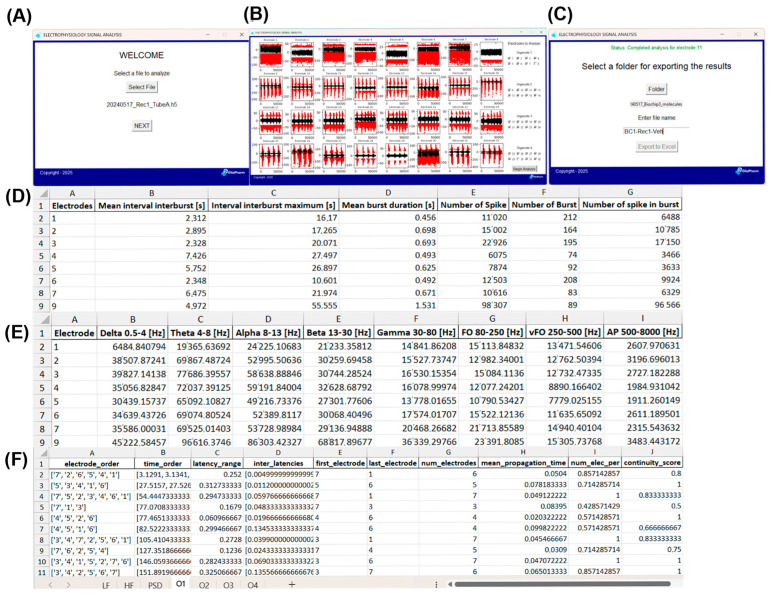
Overview of the Python-based electrophysiological analysis pipeline. (**A**–**C**) Graphical user interface of the custom analysis tool. (**A**) welcome_window: initial interface for selecting the recording file to be processed. (**B**) visu_window: visualization of the downsampled electrophysiological signal for all electrodes over the full 10 min recording (left), and interface for assigning electrodes to individual organoids via checkbox panels (right). Red dots represent detected spikes. (**C**) result_window: automated execution of the full analysis workflow in the backend and interface for choosing the export directory and output filename. (**D**–**F**) Example of an Excel output file. (**D**) Spike- and burst-related metrics, each row corresponding to an individual electrode. (**E**) PSD metrics, also reported on a per-electrode basis. (**F**) Burst-propagation metrics, with each row representing a detected propagation event. Overview of the exported Excel sheets at the bottom include low-frequency (1–40 Hz) and high frequency (500–8000 Hz) spike and burst metrics, PSD values across frequency bands, and burst-propagation data for organoids 1–4 (O1–4) within each biochip.

In this study, we developed a unified Python-based workflow that consolidates multiple independent scripts to quantify and characterize electrophysiological activity recorded from brain organoids using our MEA setup (Figure 1). Raw signals from each electrode were imported from HDF5 files and preprocessed by removing line noise followed by band-pass filtering to isolate low-frequency (1–40 Hz) and high-frequency (500–8000 Hz) components, as detailed in Section 4. PSD was computed using Welch’s method and integrated across frequency bands ranging from 0.5 to 500 Hz. To examine spectral dynamics over time, 10 min data segments were transformed into time–frequency representations using Morlet wavelets, allowing for simultaneous visualization of broadband activity and low-frequency patterns (Figure 1B). Spikes were detected independently within low and high frequency signal bands using adaptive electrode-specific thresholds derived from activity-free basal segments. Spikes were grouped into bursts using interspike interval-based criteria adapted from established MEA analysis frameworks. For each electrode, quantitative metrics including spike rates, burst counts and durations, interburst intervals, spike per burst, and band-specific PSD values were extracted and exported in a .xlsx output file (Figure 1D–F). Overall, this analytical pipeline provides a standardized, reproducible and scalable approach for analyzing electrophysiological signatures in MEA recording from brain organoids.

### 2.2. Impact of Glucose Concentration on Neuronal Activity of Brain Organoids from Healthy and GLUT1-DS Donors

Decreased glucose availability is a hallmark of GLUT1-DS and is also observed in other neurological diseases with epileptic components [10,30,31]. In brain organoids, supra-physiological glucose concentration (25 mM) is necessary to sustain survival and neuronal activity, as reported for other in vitro systems, including iPSC-derived neural cell and brain organoid models [24,32,33,34,35]. This requirement represents a divergence from in vivo physiological conditions and constitutes an inherent limitation of such in vitro models. Brain organoids were derived from iPSCs from healthy individual or a GLUT1-DS patient and cultured to maturity over a period of 12-week period, according to procedure previously described [24]. These organoids are composed of both astrocytes and neurons, including glutamatergic and GABAergic populations, as previously characterized by immunostaining and gene expression analyses [24]. The following parameters were automatically quantified: spike frequency, number of bursts, average duration of bursts, and average number of spikes per burst. In addition, PSD was quantified across several different frequency bands, including delta (0.5–4 Hz), theta (4–8 Hz), alpha (8–13 Hz), beta (13–30 Hz), gamma (30–80 Hz), fast oscillations (FO; 80–250 Hz), very fast oscillations (vFO; 250–500 Hz), and action potentials (AP; >500 Hz). Organoids were exposed for 10 min to 25 mM- or 5 mM-containing glucose media.

**Figure 2:**
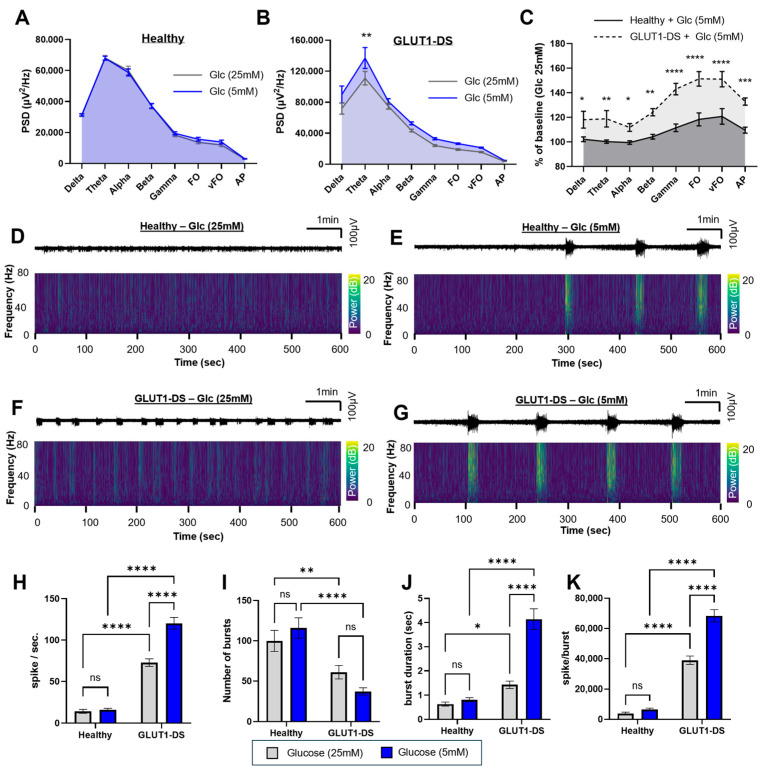
Effect of glucose on neuronal activity in brain organoids from healthy and GLUT1-DS donors. (**A**,**B**) PSD profiles comparison between brain organoids from healthy (**A**) and GLUT1-DS (**B**) origins treated with medium containing 25 mM and 5 mM glucose across frequency bands including delta (0.5–4 Hz), theta (4–8 Hz), alpha (8–13 Hz), beta (13–30 Hz), gamma (30–80 Hz), Fast Oscillations (FO, 80–250 Hz), very Fast Oscillations (vFO, 250–500 Hz) and Action Potentials (AP, >500 Hz) ranges. (**C**) PSD values in organoids from healthy and GLUT1-DS donors exposed to 5 mM glucose, expressed as a percentage of baseline activity (25 mM glucose) across all frequency bands. (**D**–**G**) Representative electrophysiological recordings over the 10 min period across the whole range of recorded frequencies (1 to 8000 Hz) (upper panels), and corresponding time frequency plots from 0 to 80 Hz (lower panels) for organoids from healthy donor treated with 25 mM glucose (**D**) or 5 mM glucose (**E**), and GLUT1-DS brain organoids treated with 25 mM glucose (**F**) or 5 mM glucose (**G**). (**H**–**K**) Quantification of the spike frequency (**H**), number of bursts (**I**), duration of bursts ((**J**), seconds) and number of spikes per burst (**K**) in brain organoids from healthy and GLUT1-DS donors after 10 min incubation in medium containing 25 mM glucose (gray bars) or 5 mM glucose (blue bars). Results are presented as mean ± SEM (*n* = 49–52 electrodes from 7 organoids of each type). Statistical analyses were performed using two-way ANOVA followed by Fisher’s post hoc test (* *p* < 0.05, ** *p* < 0.01, *** *p* < 0.001, **** *p* < 0.0001, ns, not significant).

Our data showed that glucose reduction had no detectable effect in PSD of organoids from healthy donor (Figure 2A), whereas GLUT1-DS organoids exhibited a pronounced increase in PSD following 5 mM glucose-treatment (Figure 2B), particularly in the theta bandwidth. When comparing the effect of glucose reduction (5 mM vs. 25 mM) between organoids from healthy and GLUT1-DS donors, the latter exhibited increased PSD across all frequency ranges (Figure 2C). Representative recordings of electrical activity from organoids from healthy and GLUT1-DS donors over the 10 min recording period are shown in Figure 2D–G. The upper panels display raw activity traces (1–8000 Hz), while the lower panels show time frequency plots across 1–80 Hz frequencies, with color-coded power values ranging from 0 dB (dark blue) to 20 dB (yellow).

Next, our results indicate that the number of spikes was modulated by both organoid type and glucose concentration, with a significantly higher number in GLUT1-DS compared to organoids from healthy donor, and a significantly greater increase in GLUT1-DS organoids cultured under 5 mM glucose compared to 25 mM glucose (Figure 2H). In contrast, variation in glucose did not change the number of spikes in organoids from healthy donor. The number of bursts depended only on organoid origin, with a reduced number of bursts in GLUT1-DS compared to organoids from healthy donor (Figure 2I). Similarly to the number of spikes, average burst duration was significantly affected by both genotype and treatment, showing longer bursts in organoids from GLUT1-DS donor than those from healthy donor and an additional prolongation under 5 mM glucose conditions (Figure 2J). The number of spikes per burst equally showed significant differences between organoid types and glucose changes, being elevated in GLUT1-DS organoids and further increased under 5 mM glucose conditions (Figure 2K).

Together, these results indicate that GLUT1-DS organoids display increased neuronal excitability and epileptiform features compared to organoids from healthy donor, and that reduced glucose availability exacerbates this hyperexcitability, while no significant impact of glucose concentration change was observed in organoids from healthy donor.

### 2.3. Impact of GABAergic Inhibition on Neuronal Activity of Brain Organoids from Healthy and GLUT1-DS Donors

**Figure 3:**
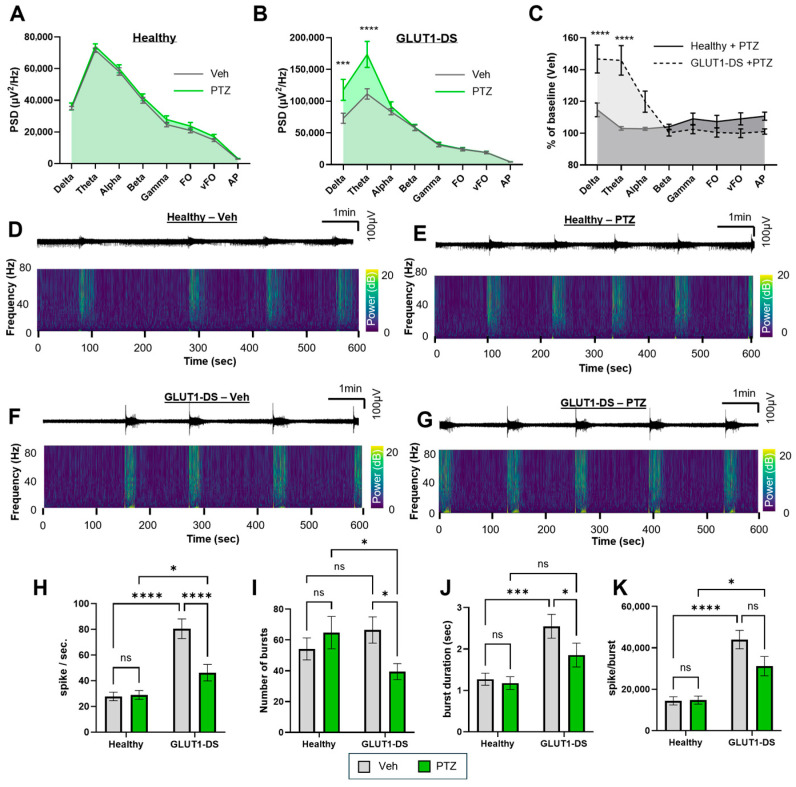
Effect of GABAergic inhibition by PTZ in brain organoids from healthy and GLUT1-DS donors. (**A**,**B**) PSD profiles comparison between brain organoids from healthy (**A**) and GLUT1-DS (**B**) origins treated with Veh (0.1% DMSO) or PTZ (3 mM) across frequency bands including delta (0.5–4 Hz), theta (4–8 Hz), alpha (8–13 Hz), beta (13–30 Hz), gamma (30–80 Hz), Fast Oscillations (FO, 80–250 Hz), very Fast Oscillations (vFO, 250–500 Hz) and Action Potentials (AP, >500 Hz) ranges. (**C**) PSD values in organoids from healthy and GLUT1-DS donor treated with PTZ, expressed as a percentage of baseline activity (Veh) across all frequency bands. (**D**–**G**) Representative electrophysiological recordings over the 10 min period across the whole range of recorded frequencies (1 to 8000 Hz) (upper panels), and corresponding time frequency plots from 0 to 80 Hz (lower panels) for organoids from healthy donor treated with Veh (**D**) or PTZ (**E**), and GLUT1-DS brain organoids treated with Veh (**F**) or PTZ (**G**). (**H**–**K**) Quantification of spike frequency (**H**), number of bursts (**I**), duration of bursts ((**J**), seconds) and number of spikes per burst (**K**) in brain organoids from healthy and GLUT1-DS donors after 10 min treatment with Veh (gray bars) or PTZ (green bars) in 25 mM glucose-containing medium. Results are presented as mean ± SEM (*n* = 33–44 from 5 to 6 organoids of each type). Statistical analyses were performed using two-way ANOVA followed by Fisher’s post hoc test (* *p* < 0.05, *** *p* < 0.001, **** *p* < 0.0001, ns, not significant).

To further evaluate epileptiform susceptibility of brain organoids from healthy vs. GLUT1-DS origin, cultures were exposed to pentylenetetrazol (PTZ), a GABA A receptor antagonist commonly used to induce seizure-like activity in experimental models [36,37]. The effects of PTZ were compared between organoids from healthy and GLUT1-DS donor (Figure 3).

Quantitative PSD analysis revealed that PTZ globally increased PSD in organoids from healthy donor, as assessed by two-way ANOVA (*p* = 0.03), although post hoc pairwise comparisons did not reach statistical significance (Figure 3A). PTZ exerted a markedly stronger increase in PSD in organoids from GLUT1-DS than from healthy donor, with significant elevations in the delta and theta frequency ranges (Figure 3B). When comparing the effect of PTZ between organoids from healthy and GLUT1-DS donor, the increase in PSD was substantially greater in GLUT1-DS, especially in the delta and theta bands (28% and 42% increase, respectively; Figure 3C). Representative traces of neuronal activity recorded during 10 min PTZ or vehicle treatment are shown in Figure 3D–G. The upper panels display raw activity signals (1–8000 Hz), while the lower panels show time frequency plots across 1–80 Hz frequencies, with color-coded power (0–20 dB). Consistent with the glucose experiments on Figure 2, the baseline number of spikes was higher in organoids from GLUT1-DS than from healthy donor. In contrast to glucose changes, PTZ exposure significantly reduced the number of spikes in GLUT1-DS, while it did not affect spike frequency in organoids from healthy origin (Figure 3H). The number of bursts was not significantly different across organoid origin or treatment (Figure 3I). Average burst duration was increased in GLUT1-DS organoids compared to those from healthy donor, but treatment did not significantly modify this parameter (Figure 3J). Consistently, the number of spikes per burst was higher in organoids from GLUT1-DS than in those from healthy donor, while PTZ did not affect this parameter (Figure 3K). Together, these findings indicate that GLUT1-DS organoids exhibit heightened sensitivity to PTZ, in particular on the PSD parameter, consistent with an underlying epileptiform predisposition.

### 2.4. Impact of Potassium Chloride (KCl)-Induced Depolarization on Neuronal Activity of Brain Organoids from Healthy and GLUT1-DS Donors

**Figure 4:**
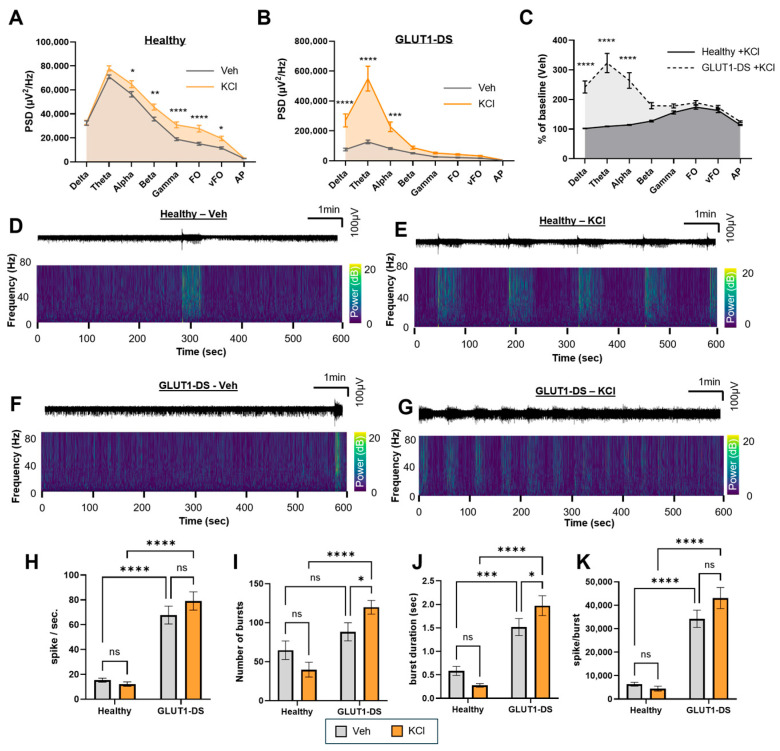
Effect of KCl-induced depolarization in brain organoids from healthy and GLUT1-DS donors. (**A**,**B**) PSD profiles comparison between brain organoids from healthy (**A**) and GLUT1-DS (**B**) origin treated with Veh (0.1% DMSO) or KCl (5 mM) across frequency bands including delta (0.5–4 Hz), theta (4–8 Hz), alpha (8–13 Hz), beta (13–30 Hz), gamma (30–80 Hz), Fast Oscillations (FO, 80–250 Hz), very Fast Oscillations (vFO, 250–500 Hz) and Action Potentials (AP, >500 Hz) ranges. (**C**) PSD values in organoids from healthy and GLUT1-DS donors treated with KCl, expressed as a percentage of baseline activity (Veh) across all frequency bands. (**D**–**G**) Representative electrophysiological recordings over the 10 min period across the whole range of recorded frequencies (1 to 8000 Hz) (upper panels), and corresponding time frequency plots from 0 to 80 Hz (lower panels) for organoids from healthy donor treated with Veh (**D**) or KCl (**E**), and GLUT1-DS brain organoids treated with Veh (**F**) or KCl (**G**). (**H**–**K**) Quantification of the spike frequency (**H**), number of bursts (**I**), duration of bursts ((**J**), seconds) and number of spikes per burst (**K**) in brain organoids from healthy and GLUT1-DS donor after 10 min-treatment with Veh (gray bars) or KCl (orange bars) in 25 mM glucose-containing medium. Results are presented as mean ± SEM (*n* = 27–44 electrodes from 5 to 6 organoids of each type). Statistical analyses were performed using two-way ANOVA followed by Fisher’s post hoc test (* *p* < 0.05, ** *p* < 0.01, *** *p* < 0.001, **** *p* < 0.0001, ns, not significant).

To assess neuronal responsiveness to global depolarization, organoids were treated with KCl to induce membrane depolarization and enhance network activity. As shown in Figure 4, PSD analyses revealed significant differences across conditions. KCl induced a global increase in PSD in organoids from healthy donor with significance in the alpha through vFO bands (Figure 4A). Similarly, PSD was increased by KCl in GLUT1-DS organoids, significantly in the delta to alpha bands (Figure 4B). Comparison of KCl-induced changes between organoid types demonstrated a significantly greater response to KCl in organoids from GLUT1-DS than those from healthy origin, driven mainly by increases in the delta, theta, and alpha frequencies (137%, 196% and 132% increases, respectively; Figure 4C). Representative 10 min recordings of neuronal activity with or without KCl are shown in Figure 4E–H. The upper panels display raw electrical activity traces (1–8000 Hz), and the lower panels show time frequency plots across 1–80 Hz frequencies (0–20 dB). Similarly to experimental conditions presented above, the number of spikes was higher in organoids from GLUT1-DS than from healthy donors under basal conditions, while KCl treatment itself had no effect on this parameter (Figure 4H). The number of bursts was not different between Veh-treated groups but was significantly increased in organoids from GLUT1-DS compared to those from healthy origin treated with KCl (Figure 4I). Average burst duration was also elevated in GLUT1-DS organoids compared to those from healthy donor, while KCl did not modulate this parameter (Figure 4J). The number of spikes per burst also remained higher in organoids from GLUT1-DS than in those from healthy origin but were not sensitive to the treatment (Figure 4K). These data indicate that while both organoid types respond to depolarizing stimulation, GLUT1-DS organoids exhibit amplified and broadband neuronal activation, in particular on the PSD parameter, consistent with intrinsic network hyperexcitability.

### 2.5. Specificity of Neuronal Activity Signal Assessed with Tetrodotoxin (TTX)

**Figure 5:**
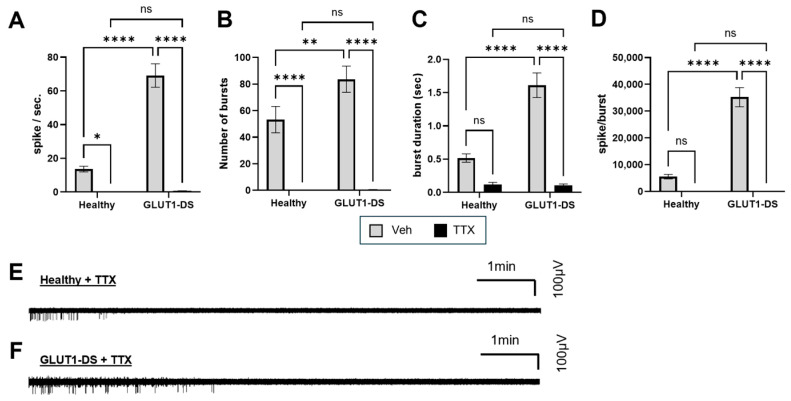
Effect of neuronal activity inhibition by TTX in brain organoids from both healthy and GLUT1-DS donors. (**A**–**D**) Quantification of spike frequency (**A**), number of bursts (**B**), duration of bursts ((**C**), seconds) and number of spikes per burst (**D**) in brain organoids from healthy and GLUT1-DS origins after 10 min treatment with Veh (0.1% DMSO, gray bars) or TTX (200 nM, black bars) in 25 mM glucose-containing medium. (**E**,**F**) Representative electrophysiological recordings over a 10 min period across the whole range of recorded frequencies (0.5 to 500 Hz) for organoids from healthy (**E**) or GLUT1-DS (**F**) donor treated with TTX. Results are presented as mean ± SEM (*n* = 36–44 from 5 to 6 organoids of each type). Statistical analyses were performed using two-way ANOVA followed by Fisher’s post hoc test (* *p* < 0.05, ** *p* < 0.01, **** *p* < 0.0001, ns, not significant).

Finally, to verify that the recorded electrical activity originated from neuronal firing, organoids from both healthy and GLUT1-DS donors were treated with TTX, a potent voltage-gated sodium channels blocker. As expected, TTX markedly reduced neuronal activity across all parameters, including the number of spikes (Figure 5A), number of bursts (Figure 5B), average burst duration (Figure 5C), and number of spikes per burst (Figure 5D). Representative activity traces illustrate a rapid decline in firing frequency within minutes after TTX application, followed by complete silencing of electrical activity (Figure 5E,F). These results confirm that the electrophysiological signals recorded from brain organoids from both healthy and GLUT1-DS donors reflect genuine neuronal activity.

## 3. Discussion

Our results demonstrate that our newly developed electrophysiological analysis pipeline (Figure 1) enables characterization of epileptiform network activity in human brain organoids recorded on an MEA platform. We show that human brain organoids recapitulate disease-relevant phenotypes related to glucose restriction and neuronal excitability, which were selectively amplified in organoids derived from a GLUT1-DS patient. Quantitatively, GLUT1-DS organoids exhibited increased spike frequency, prolonged bursts and increased number of spikes per burst than organoids from healthy donor (Figure 2). Reduction in glucose concentration from 25 mM to 5 mM exacerbated the higher number of spikes, duration of bursts and number of spikes per burst in GLUT1-DS. Similarly, it significantly raised PSD in GLUT1-DS with a prominent effect in theta wavelength, whereas organoids from healthy donor were largely unaffected by glucose changes. These data indicate that neuronal activity, which is exemplified through the number of spikes, duration of bursts and number of spikes per burst, is higher in GLUT1-DS, and exacerbated by reduced availability of glucose (Figure 2). In line with this observation, PSD was increased along all measured frequencies in GLUT1-DS organoids compared to those from healthy donor, which is also exacerbated by reduced glucose availability. Most pronounced effect of reduced glucose availability in GLUT1-DS occurred in the theta frequency band, which abnormal oscillations and pre-ictal synchronization in limbic and cortical circuits are associated with epileptiform activity [38,39,40]. While organoids lack vasculature and endothelial GLUT1, the finding that GLUT1-DS exhibit hyperactivity compared to cultures from healthy donor indicates that astrocytes, which express GLUT1, are responsible to mediate this hypometabolism–hyperexcitability coupling.

The selective vulnerability of GLUT1-DS organoids to reduced glucose supports a mechanistic link between hypometabolism and hyperexcitability. Although reduced energy substrate availability might be expected to dampen neuronal activity, clinical and experimental evidence implies the opposite at the network level. In temporal lobe epilepsy, hypometabolism measured with ^18^F-FDG PET reliably co-localizes with seizure-onset zones and is associated with improved seizure control following resection [10]. Beyond this spatial correspondence, quantitative analyses of mesial temporal hypometabolism have demonstrated predictive utility at the individual level, enabling patient-specific classification of seizure-free versus non–seizure-free outcomes prior to surgery [31]. Mechanistic reason is that maintaining asynchronous network demands significant energy, as both excitatory and inhibitory systems must be sustained, while local or global synchronization of electrical events leads to uniform neuronal discharges resulting in epileptic seizures [41]. Similar metabolic deficiencies are related to network hyperactivity and synchronization in pathological contexts beyond epilepsy. In Alzheimer’s disease (AD), hyperexcitability, seizures and cerebral hypometabolism exacerbate one another, accelerating cognitive decline [30]. Network hypersynchrony and disruptions in oscillatory rhythms correlate with cognitive impairment in AD and have been detected decades prior to clinical symptom onset [42]. Clinically, multiple studies report an increased incidence of seizures in individuals with mild to moderate AD [43,44,45]. Converging evidence further suggests that these hyperexcitable and hypersynchronous network states arise from dysfunction within interneuron-dependent inhibitory circuits [46,47,48].

Our results next indicate that the sensitivity of GLUT1-DS to changes that trigger epileptiform activity generalizes across other pharmacological perturbations, such as treatment with pentylenetetrazol (PTZ; Figure 3) and with potassium chloride (KCl; Figure 4). Pro-epileptic effect of PTZ comes from its allosteric interaction to GABA A receptors, thereby reducing the affinity of the receptor to its natural ligand GABA, and the inhibitory effect of GABAergic neurons on a network level [37,49]. Both organoid types were shown to express glutamatergic and GABAergic markers [24]. Our data indicates that treatment with PTZ dramatically enhanced PSD signal across delta and theta bands in GLUT1-DS organoids, while it only marginally affected organoids from healthy donor (Figure 3A–G). In contrast to the data resulting from changes in glucose concentration, treatment with PTZ led to significant reduction in the number of spikes, number of bursts, burst duration and spike per bursts in GLUT1-DS (Figure 3H–K). These results highlight the similarities (PSD) and differences (spike and bursts frequencies) between reduced glucose availability and GABAergic inhibition in the modulation of neuronal activity of brain organoids. Interestingly, PSD analyses highlighted significant impact of treatment and organoid origin in the theta power, which is aligned with model of status epilepticus in kainic acid-treated rodents, where dynamic changes in theta band activity have been associated with seizure severity, progression toward convulsive states, and large-scale network hypersynchrony during epileptiform activity [50].

Investigation of the role of KCl revealed that GLUT1-DS organoids were also more sensitive to global depolarization. KCl had significant impact on PSD in organoids from both healthy and GLUT1-DS donors, but stronger impact in delta, theta and alpha bands when comparing organoids from healthy to GLUT1-DS origin (Figure 4A–G). KCl equally increased the spike frequency, the number of bursts and the duration of bursts in GLUT1-DS organoids, but not in those from healthy donor (Figure 4H,I). Apart from the intrinsic sensitivity of neuronal depolarization caused by hypometabolic nature of GLUT1-DS, impaired glucose uptake in astrocytes can weaken the capacity of astrocytes to buffer K^+^ and clear glutamate during heightened neuronal activity [7,9]. The resulting extracellular K^+^ and glutamate accumulations could further amplify neuronal hyperactivity mediated by KCl. Finally, treatment with TTX uniformly reduced neuronal activity across all metrics in both organoid types, confirming that our readouts track voltage-gated Na^+^ channel-dependent neuronal activity (Figure 5).

In conclusion, human brain organoids capture the key pathophysiological coupling between hypometabolism and hyperexcitability in GLUT1-DS. Through genotype-selective responses to glucose reduction, GABAergic challenge by PTZ and general depolarization by KCl, our data highlights an elevated sensitivity of GLUT1-DS to epileptiform challenges. The results argue that intrinsic predisposition in GLUT1-DS organoids converts metabolic or GABAergic challenges into epileptiform hyperactivity. Furthermore, our data converges with studies in focal epilepsy showcasing the link between hypometabolism and neuronal hyperactivity, presumably through neural network hypersynchrony [10,51]. Such brain organoid platform that assesses neuronal activity and epileptiform parameters provides a tractable, human-specific system to mechanistically dissect and pharmacologically correct this coupling. As this study is limited to the organoids derived from one healthy donor and one GLUT1-DS patient with distinct genetic backgrounds, future work should incorporate iPSC lines from multiple healthy donors and GLUT1-DS patients carrying different mutations. Such human cell-based models integrating organoids from diverse controls and patients could accelerate the discovery of metabolic rescue strategies (e.g., enhancing astrocytic glycolysis, alternative fuels, GLUT1 trafficking) and support individualized patient diagnosis and characterization of brain metabolic profile.

## 4. Materials and Methods

### 4.1. Generation of Brain Organoids

Brain organoids from a healthy donor or a GLUT1-DS patient were generated according to the procedure previously described [24]. Induced pluripotent stem cells (iPSCs) were acquired from the Coriell Institute, including control cells from a healthy donor (GM28404) and GLUT1-DS patient-derived cells carrying an SLC2A1 mutation (c.1454C>T; GM27896) [52]. After thawing, iPSCs were plated in laminin-coated dishes and maintained in mTeSR plus medium for 7 days until they reached 80–90% confluence. Next, differentiation into neural stem cells (NSCs) began with a passage in a Geltrex 60 mm coated dish at a seeding of 40,000 cell/cm^2^ density. For another 7 days, cells were cultured in Neurobasal medium with neural induction supplements. NSCs were then expanded in a medium composed of half Neurobasal medium and half knockout DMEM/F-12 basal medium with GlutaMAX and neural induction supplement, until they reached maturity after approximately 7 days. NSCs were then seeded in 6-well plates (250,000 cells/well) and maintained under constant rotation (80 rpm) at 37 °C in NSC expansion medium. After one week of sphere formation, organoids were differentiated in DIFF1 medium (week 2) containing BDNF (20 ng/mL), GDNF (20 ng/mL), dibutyryl cyclic AMP (500 µM) and 2-phospho-ascorbic acid (200 µM), followed by DIFF2 medium (weeks 3–5), a 1:1 mixture of DIFF1 and Neuron Differentiation Medium (NDM). Maturation proceeded in NDM until week 8.

### 4.2. Electrophysiological Recordings

When they reached maturity after differentiation, brain organoids were transferred to air–liquid interface (ALI) culture for two days and then placed on custom microelectrode array biochips for 5 days. The platform featured automated perfusion (0.53 mL/min) and real-time signal acquisition using the SpikeOnChip (SPOC) system [53]. Each biochip contained four recording sites with eight recording electrodes and three reference electrodes per site, sampling at 30 kS/s with 16-bit resolution.

### 4.3. Brain Organoid Treatments

Brain organoids were maintained in DMEM medium containing 25 mM glucose for one to two days before treatment. During treatment, the electrical activity was first recorded in DMEM medium containing 25 mM glucose supplemented with vehicle (0.1% DMSO) for 10 min, serving as activity baseline. The medium was then replaced by stimulation medium for 10 min with ongoing electrical activity recording. Stimulation medium consisted of DMEM with 25 mM glucose supplemented with potassium chloride (KCL, 5 mM), tetrodotoxin (TTX, 200 nM), or pentylenetetrazol (PTZ, 3 mM), or DMEM with 5 mM glucose supplemented with vehicle (0.1% DMSO).

### 4.4. Custom Graphical Interface for MEA Data Analyses

A custom graphical user interface (GUI) was developed in Python to facilitate control over the processing and interpretation of MEA recordings designed to handle HDF5 files generated by the SPOC GUI platform [54]. Data output was organized into three categories: raw signal, spike detection, and local field potential (LFP) analysis. All GUI windows in the application shared a unified layout, defined in a common base file (base_window.py). After launching via an executable file that redirected to the main entry point (main.py), the analysis workflow was divided into three sequential GUI windows, as shown in Figure 1. First, welcome_window.py allowed the user to select the HDF5 file for analysis. Second, visu_window.py displayed raw electrophysiological signals from 32 electrodes. A panel of 32 checkboxes allowed the user to select specific electrodes for further analysis. Third, result_window.py automatically initiated signal processing for the selected electrodes. Upon completion, the results were exported into .xlsx format for analysis. Electrophysiological analyses were conducted using a custom Python-based pipeline designed to process high-resolution extracellular recordings obtained from multiple electrodes. All codes were executed using Python 3.10, leveraging libraries such as NumPy (v1.26), SciPy (v1.12), pandas (v2.2), and h5py (v3.11). The analysis comprised several preprocessing, filtering, feature extraction, and summarization steps, as described below. Software code is accessible on https://github.com/LengaCodes/Electrophy_Analysis_Tool_GP (accessed on 20 November 2025).

### 4.5. Signal Processing

The signal was first processed through a notch filter centered at 50 Hz to remove background noise, using the scipy.signal.iirnotch function, with a quality factor of 30. The signal next underwent two successive Butterworth band-pass filtering steps to isolate relevant frequency components. Specifically, a low-frequency (LF) band filter ranging from 1 to 40 Hz and a high-frequency (HF) band filter ranging from 500 to 8000 Hz were implemented. Both filters employed second order forward-backward filtering, using the scipy.signal.butter and scipy.signal.filtfilt functions, to preserve phase integrity.

### 4.6. Power Spectral Density (PSD) and Time Frequency Plot Analyses

PSD was computed using Welch’s method (scipy.signal.welch) on the notch-filtered signal. The signal was divided into 2 s segments (nperseg = 2 × sampling_rate), and the resulting power spectrum was integrated across eight frequency bands, delta (0.5–4 Hz), theta (4–8 Hz), alpha (8–13 Hz), beta (13–30 Hz), gamma (30–80 Hz), FO (80–250 Hz), vFO (250–500 Hz), and AP (>500 Hz). For each frequency band, mean spectral power was computed and converted from units of V^2^/Hz to mV^2^/Hz. The low-frequency trace was down-sampled from 30 kHz to 100 Hz for time–frequency decomposition. Continuous wavelet transformation with a Morlet kernel covered 1–80 Hz, and power was computed as the squared modulus of coefficients. Median power at each frequency served as baseline, with results expressed in decibels (dB). Time frequency plots included broadband and low-frequency traces aligned with a color-coded spectrogram (1–80 Hz, power range 0–20 dB).

### 4.7. Spike and Bursts Detections

Spike activity was analyzed withing the HF-filtered band recorded from each of the 32 electrodes. For each dataset, detection threshold was calculated from signal segments identified as activity-free, providing electrode-specific adaptation to baseline noise. To identify activity-free background signal, a moving window of 0.5 s duration was scanned across the notch-filtered signal, and a segment was considered activity-free if all values remained below a fixed amplitude threshold of 35 mV, consistent with the threshold used in visu_window.py. The threshold was set to 4.5× the background noise signal standard deviation for LF and 6× the background noise signal standard deviation for HF [24]. A spike was detected when a local extremity, either positive or negative, exceeded the corresponding threshold and was greater than its immediate neighbors. Detected events were stored as the time point and signal amplitude of the identified extremity.

Detected spikes were grouped into bursts based on interspike interval criteria. In the LF domain, a minimum of 5 spikes separated by ≤0.3 s between each other were assigned to the same burst, while in the HF domain, the maximum allowable interval between spikes was 0.05 s, according to procedure previously published [24]. Burst were merged when consecutive bursts were separated by less than 2.5 s in the LF domain or 0.5 s in the HF domain. For each burst, quantitative parameters including its duration, interburst interval, and the number of constituent spikes were calculated. Spikes that occurred within a burst were isolated to calculate burst spike counts, as opposed to spikes occurring outside of bursts activity.

### 4.8. Statistical Analyses

Statistical significance was calculated using 2-way analysis of variance (ANOVA) followed by Tukey’s post hoc test for pair-wise comparisons. Outliers were detected using ROUT’s test and removed from the analysis. GraphPad Prism v10 was used for all statistical analyses. *, **, *** and **** refer to *p* values of <0.05, 0.01, 0.001 and 0.0001, respectively. The total number of replicates (*n*) is indicated in the figure legends.

## Data Availability

The data presented in this study are available on request from the corresponding author. (The raw data supporting the conclusions of this article are not publicly available due to privacy restrictions). Software code is accessible on https://github.com/LengaCodes/Electrophy_Analysis_Tool_GP (accessed on 20 November 2025).

## Abbreviations

The following abbreviations are used in this manuscript:

AD	Alzheimer’s Disease
AMP	Adenosine monophosphate
ANLS	Astrocyte–neuron lactate shuttle
ALI	Air–liquid interface
ANOVA	Analysis of variance
BDNF	Brain-derived neurotrophic factor
dB	decibel
DMSO	Dimethylsulfoxide
^18^F-FDG PET	^18^F-fluorodepxyglucose positron emission tomography
GDNF	Glial cell line-derived neurotrophic factor
GLUT1	Glucose Transporter 1
GLUT1-DS	Glucose Transporter 1-Deficiency Syndrome
GUI	Graphical User Interface
HF	High frequency
Hz	Hertz
iPSC	Induced-pluripotent stem cell
KCl	Potassium Chloride
LF	Low frequency
LFP	Local Field Potential
MEA	Multielectrode array
MCT	Monocarboxylate transporter
NDM	Neuron Differentiation Medium
NSC	Neural stem cell
PSD	Power Spectral Density
PTZ	Pentylenetetrazol
SPOC	SpikeOnChip
TLE	Temporal lobe epilepsy
TTX	Tetrodotoxin
